# Engineering of a green-light inducible gene expression system in *Synechocystis* sp. PCC6803

**DOI:** 10.1111/1751-7915.12098

**Published:** 2013-12-12

**Authors:** Koichi Abe, Kotone Miyake, Mayumi Nakamura, Katsuhiro Kojima, Stefano Ferri, Kazunori Ikebukuro, Koji Sode

**Affiliations:** 1 Department of Biotechnology and Life Science, Tokyo University of Agriculture & Technology2-24-16 Naka-cho, Koganei, Tokyo, 184-8588, Japan; 2 JST, CREST2-24-16 Naka-cho, Koganei, Tokyo, 184-8588, Japan

## Abstract

In order to construct a green-light-regulated gene expression system for cyanobacteria, we characterized a green-light sensing system derived from *Synechocystis* sp. PCC6803, consisting of the green-light sensing histidine kinase CcaS, the cognate response regulator CcaR, and the promoter of *cpcG2* (P_cpcG__2_). CcaS and CcaR act as a genetic controller and activate gene expression from P_cpcG__2_ with green-light illumination. The green-light induction level of the native P_cpcG__2_ was investigated using GFPuv as a reporter gene inserted in a broad-host-range vector. A clear induction of protein expression from native P_cpcG__2_ under green-light illumination was observed; however, the expression level was very low compared with P_trc_, which was reported to act as a constitutive promoter in cyanobacteria. Therefore, a Shine-Dalgarno-like sequence derived from the *cpcB* gene was inserted in the 5′ untranslated region of the *cpcG2* gene, and the expression level of CcaR was increased. Thus, constructed engineered green-light sensing system resulted in about 40-fold higher protein expression than with the wild-type promoter with a high ON/OFF ratio under green-light illumination. The engineered green-light gene expression system would be a useful genetic tool for controlling gene expression in the emergent cyanobacterial bioprocesses.

## Introduction

Cyanobacteria are considered an ideal host for the production of biofuel or biomaterials, thanks mainly to their ability to directly convert carbon dioxide to the target substance, requiring only sunlight, water and some inorganic compounds. Because most cyanobacteria are transformable, they can be genetically engineered for the efficient production of biofuel and biomaterials. For this purpose, a number of researchers have been attempting to construct biosynthetic pathways using various genetic components from *Escherichia coli* or other organisms (Atsumi *et al*., 2009; Liu and Curtiss, 2009; 2012; Oliver *et al*., 2013). However, some of the promoters are not compatible with cyanobacteria (Huang *et al*., 2010) presumably because of differences in the RNA polymerase from *E. coli* and cyanobacteria (Schneider and Hasekorn, 1988). Moreover, conventionally reported bacterial gene expression systems are based on the induction by specific chemicals, such as isopropyl β-D-1-thiogalactopyranoside (IPTG) and metal ions (Briggs *et al*., 1990; Geerts *et al*., 1995; Lopez-Maury *et al*., 2002), which are not practical considering the large-scale cultivation of the cyanobacterial process. In addition, chemical inducers are difficult to remove from the culture medium, making them unsuitable for the downstream water recycling process. Therefore, an alternative gene expression system specific for the cyanobacterial bioprocess should be developed.

Cyanobacteria have various light-sensing systems to regulate an effective photosynthesis or avoid photodamage by strong or short-wavelength light. Light-sensing systems regulate the gene expression, the enzymatic activity for second messenger production, or the phototaxis response upon illumination with various lights, such as UV light (Narikawa *et al*., 2011; Song *et al*., 2011), blue light (Yoshihara *et al*., 2004), green light (Terauchi *et al*., 2004; Hirose *et al*., 2008; 2010) and red light (Yeh *et al*., 1997; Terauchi *et al*., 2004). Most sensing systems are based on a two-component regulatory system consisting of a sensor histidine kinase and a cognate response regulator. Absorption of light induces a conformational change of the sensing protein and activates its kinase activity to transfer the signal to the cognate response regulator. Phosphorylated response regulator binds to the upstream region of a promoter, resulting in induction of the gene expression or binds to the protein controlling the movement of the flagella.

The expression of the phycobilisome linker gene *cpcG2* has been reported to be chromatically regulated by the sensor histidine kinase CcaS and the cognate response regulator CcaR in *Synechocystis* sp. PCC6803 (Hirose *et al*., 2008). CcaS catalyses autophosphorylation followed by phosphotransfer to CcaR under green light and dephosphorylation of CcaR under red light.

We aimed to construct a gene expression system regulated in cyanobacteria by green light, which is not a major light source for photosynthesis. In order to construct a green-light-regulated gene expression system for cyanobacteria, we utilized and engineered a green-light sensing system derived from *Synechocystis* sp. PCC6803, consisting of the green-light sensing histidine kinase CcaS, the cognate response regulator CcaR, and P_cpcG2_. CcaS and CcaR act as a genetic controller and activate gene expression from P_cpcG2_ with green-light illumination.

## Results and discussion

The P_cpcG2_ promoter was cloned from *Synechocystis* sp. PCC6803 and inserted upstream of the gene encoding the GFPuv reporter protein on the broad-host-range vector pKT230 (Bagdasarian *et al*., 1981) as described in Supplementary information (Table S1). *Synechocystis* sp. PCC6803 was transformed with the resulting plasmid and precultured under red-light illumination. The cultures were then incubated with continuous illumination with only green light, only red light, or with both green and red light together, and the fluorescence intensity of the cultures were measured.

**Figure 1:**
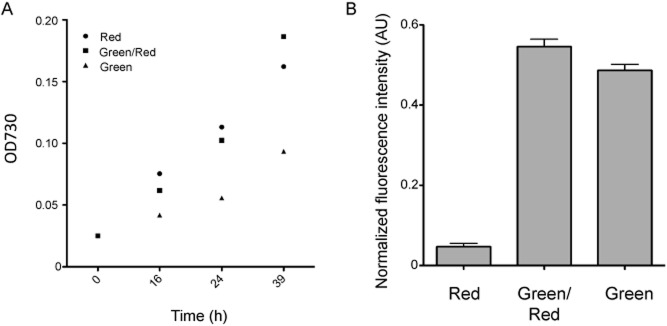
Control of gene expression by green light in *Synechocystis* sp. PCC6803 harbouring pKT230-P_cpcG__2_-GFPuv.A. Growth curve under red (20 μmol m^−2^ s^−1^), green (20 μmol m^−2^ s^−1^), or both green and red light (green/red). Growth was determined by measuring the optical density at 730 nm.B. Fluorescence intensity of cells was normalized by their respective optical density at 730 nm after 39 h of incubation under each light illumination. After each culture was washed by phosphate buffered saline, fluorescence intensity was measured by a plate reader (λex. 395 nm/λem. 520 nm) (Thermofisher Scientific, Waltham, MA)

We observed an increase in the fluorescence intensity derived from GFPuv under not only green light but also green-and red-light illumination. These increased fluorescence levels were nearly identical when normalized according to the culture's optical density (OD) at 730 nm (Fig. 1). It has been reported that red-light illumination represses CcaS, resulting in dephosphorylation of CcaR by CcaS phosphatase activity (Hirose *et al*., 2008). However, our results indicate that CcaS can be activated by green-light illumination, thus inducing transcription by P_cpcG2_, even under simultaneous illumination with red light, which is necessary for efficient cell growth. However, the expression level estimated by the GFPuv fluorescence intensity was quite low compared with the gene expression using the P_trc_ promoter (Abe *et al*., 2001).

**Figure 2:**
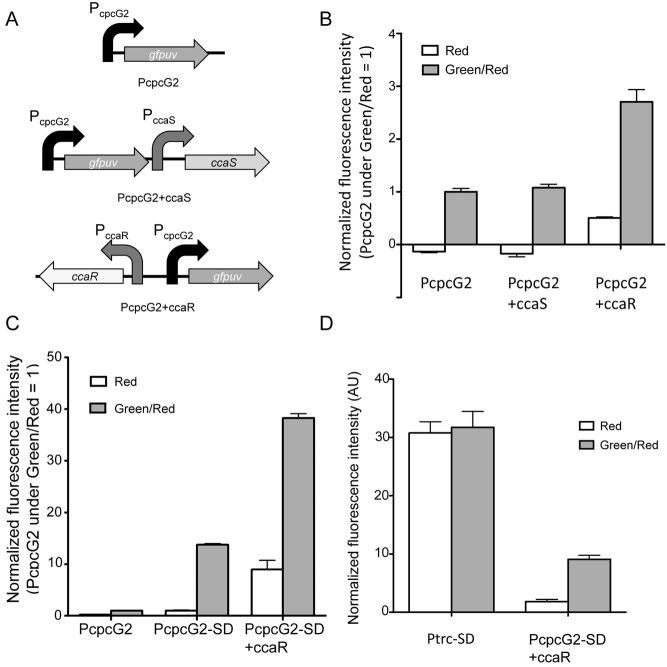
Evaluation of engineered green-light sensing system in *Synechocystis* sp. PCC6803 under red or green/red light.A. Schematic representation of each construct introduced into pKT230: P_cpcG__2_-GFPuv (PcpcG2), P_cpcG__2_-GFPuv-P_ccaS_-ccaS (PcpcG2+*ccaS*), P_cpcG__2_-GFPuv-P_ccaR_-*ccaR* (PcpcG2+*ccaR*).B. Relative normalized fluorescence intensity of *Synechocystis* sp. PCC6803 harbouring PcpcG2, PcpcG2+*ccaS*, or PcpcG2+*ccaR* under red or green/red light. Each value is normalized to cell optical density and provided relative to the normalized value of PcpcG2 under green light (set as 1).C. Relative normalized fluorescence intensity of *Synechocystis* sp. PCC6803 harbouring PcpcG2, pKT230-P_cpcG__2_-SD-GFPuv (PcpcG2-SD), P_cpcG__2_-SD-GFPuv+*ccaR* (PcpcG2-SD+*ccaR*) under red or green/red light. Each value is normalized to cell optical density and provided relative to the normalized value of PcpcG2 under green light (set as 1).D. Relative normalized fluorescence intensity of *Synechocystis* sp. PCC6803 harbouring pKT230-P_trc_-SD-GFPuv (Ptrc-SD), whose SD-like sequence was replaced by the SD-like sequence of *cpcB*, and P_cpcG__2_-SD+*ccaR*. Each value is normalized to cell optical density.

Attempting to improve the gene expression level, we first focused on the gene dosage effect of CcaS on the GFPuv expression level. In *Synechocystis* sp., PCC6803 that are continuously illuminated with green and red light together, CcaR might not all be phosphorylated by CcaS. We investigated whether increasing the amount of CcaS in *Synechocystis* sp. PCC6803 would result in an increased CcaR phosphorylation level in response to green-light illumination, consequently increasing the GFPuv expression level. To increase CcaS activity by increasing its expression level, the plasmid harbouring P_cpcG2_-GFPuv and the *Synechocystis* sp. PCC6803 *ccaS* gene under its native promoter (Fig. 2A) were constructed (Table S1). Additional expression of CcaS showed nearly identical GFPuv-derived fluorescence intensity to that expressing only the endogenous CcaS (Fig. 2B). CcaS that is activated by green light would show kinase activity towards CcaR, while CcaS that is repressed by red light would instead show dephosphorylation activity towards CcaR. Our results suggest that increasing the amount of CcaS expression has not changed the kinase to dephosphorylation activity ratio, resulting in unchanged level of CcaR phosphorylation.

The gene dosage effect of CcaR on the GFPuv expression level was then investigated. Considering that the green-light gene expression system utilized endogenous CcaR, it may not be sufficient to fully activate the expression of exogenously introduced P_cpcG2_, which is encoded in the broad-host-range vector pKT230, whose copy number greater than 10 (Barth and Grinter, 1974). A plasmid harbouring P_cpcG2_-GFPuv and the *Synechocystis* sp. PCC6803 *ccaR* gene under its native promoter (Fig. 2A) was therefore constructed (Table S1), and its GFPuv expression level was investigated under green-and red-light illumination. As a result, an almost three-fold increase of GFPuv-derived fluorescence intensity under green-and red-light illumination was observed compared with cells only expressing the endogenous CcaR (Fig. 2B). These results indicate that the induction level by the green-light gene expression system was improved by increasing the CcaR expression level. However, an increase in fluorescence intensity was also observed under red-light illumination in the absence of green light possibly because of cross-talk of some endogenously existing histidine kinases catalysing the phosphorylation of exogenously overexpressed CcaR. Additionally, some response regulators have been reported to bind and activate a corresponding promoter under unphosphorylated condition (Schar *et al*., 2005; Kato *et al*., 2008). Therefore, unphosphorylated CcaR may contribute to the background expression under red-light illumination.

Protein expression level is mainly controlled by a combination of promoter activity, strength of ribosome binding site (RBS), mRNA structure around the RBS and start codon, and some *trans* elements such as antisense RNA. The P_cpcG2_ promoter does not have a typical Shine-Dalgarno (SD) sequence upstream of the start codon. In fact, most cyanobacterial genes do not have SD-like sequence, and only 26% of the genes harbour an SD-like sequence at the optimal position (Ma *et al*., 2002). For some of these genes that do not have an SD-like sequence, translation is initiated by recognition of the 5′-untranslated region (UTR) by the S1 ribosomal protein, which recognizes an AU-rich sequence upstream of the start codon (Tzareva *et al*., 1994). As the upstream region of P_cpcG2_ has several successive AU bases (5′-UUAAGUUUAAUUACUAACUUUAUCU-3′), *cpcG2* expression might also be regulated by the S1 ribosomal protein. Furthermore, using the RNA secondary structure prediction software Mfold (Zuker, 2003), a stable secondary structure could not be predicted in the region. Considering that the observed weak expression of GFPuv under P_cpcG2_ may be due to the lack of a SD-like sequence, we evaluated the effect of adding an SD sequence on the gene expression under P_cpcG2_.

We searched for SD-like sequence candidates from 5′-UTRs of cyanobacterial genes. Some photosynthesis-related proteins are highly expressed in cyanobacteria, such as the *psbA*-encoded D1 protein and the *cpcB*-encoded c-phycocyanin, which have in their gene's 5′-UTR the SD-like sequences AGGA and AGGAG respectively. Furthermore, the SD-like sequence from *cpcB* is completely complementary to the 3′ region of 16S rRNA of *Synechocystis* sp. PCC6803 and is highly conserved among various cyanobacteria. We therefore considered that the SD-like sequence of the *cpcB* gene would function strongly in *Synechocystis* sp. PCC6803. The SD-like sequence derived from the *cpcB* gene of *Synechococcus* sp. PCC7002, 5′-UAUAAGUAGGAGAUAAAAAC-3′, was introduced upstream of the start codon of the GFPuv gene under the P_cpcG2_ (Table S1).

The addition of the SD-like sequence to P_cpcG2_ resulted in about 15-fold higher GFPuv-derived fluorescence intensity when illuminated with both red and green light (P_cpcG2_-SD in Fig. 2C). Quantitative reverse transcription polymerase chain reaction analysis revealed that the P_cpcG2_-SD culture contained twice the amount of mRNA of GFPuv than P_cpcG2_ (data not shown) probably because of the enhanced ribosome binding leading to stabilization of the mRNA. The increase in GFPuv fluorescence is therefore mainly attributable to an increase in translation efficiency rather than transcription. The AU-rich sequence upstream of SD sequence improved the expression level in *Escherichia coli* (Komarova *et al*., 2002). We therefore consider that the introduction of an SD-like sequence showed a synergetic effect with the AU-rich sequence on protein translation.

We then constructed a pKT230-derived vector harbouring P_cpcG2_-SD-GFPuv and *ccaR* under the control of its native promoter (Table S1). We expected to observe a cumulative effect by combining the enhanced transcription from the additional exogenously expressed CcaR with the enhanced translation from the introduction of an SD-like sequence. Under red and green illumination, the constructed vector yielded about 40-fold greater GFPuv-derived fluorescence intensity than cells harbouring the plasmid expressing GFPuv without the SD-like sequence and without the exogenous CcaR (Fig. 2C). This result corresponds to an almost direct cumulative effect of the almost threefold increase from the exogenous CcaR (Fig. 2B) and the approximately 15-fold increase from the introduction of the SD-like sequence (Fig. 2C). The constructed vector also showed threefold lower GFPuv expression level than under the P_trc_ promoter, which also has an SD-like sequence (Fig. 2D). However, because the P_trc_ promoter shows very poor ON/OFF ratio in *Synechocystis* sp. PCC6803 with IPTG as inducer (Huang *et al*., 2010), P_cpcG2_-SD+*ccaR* is an attractive alternative for an inducible gene expression system for *Synechocystis* sp. PCC6803.

Inducible promoters are important genetic tools to elucidate gene function and to effectively control protein production. However, there is little available information on inducible promoters for cyanobacteria. Some of the conventional inducible promoters used in *E. coli*, such as the lactose promoter or its derivatives, do not work well in *Synechocystis* sp. PCC6803 (Huang *et al*., 2010) possibly because of structural differences in the RNA polymerase (Schneider and Hasekorn, 1988). Guerrero and colleagues (2012) reported that the P_A1lacO1_ promoter showed better induction in *Synechocystis* sp. PCC 6803 by IPTG compared with the P_trc_ promoter. However, synthetic chemical inducers are not cost-effective, even for IPTG, which is widely used for *E. coli*. Although cyanobacteria have many metal ion inducible promoters, such as the nickel-inducible nrsB promoter, which shows high ON/OFF ratio (Lopez-Maury *et al*., 2002), toxic heavy metal ions are not suitable for large-scale production of biofuel or biomaterials. The light-inducible psbA1 and psbA2 promoters, which have also been used for controlling protein expression in cyanobacteria, require dark cultivation before induction (Agrawal *et al*., 2001). In this study, to control gene expression, we engineered a green-light sensing system that showed a high protein expression level with a high ON/OFF ratio. Because green light is not essential for photosynthesis, we can cultivate cyanobacteria with optimal growth before gene induction. During green-light illumination, we expected that the simultaneous illumination with red and green light would cancel each other out, resulting in very low activation of target gene expression because CcaS, which has a phycocyanobilin chromophore, is reported to be converted to an activated state by absorbing green light and converted to a repressed state by red light (Hirose *et al*., 2008). However, we found that green-light illumination significantly activated target gene expression, even with simultaneous red-light illumination. This green-light sensing system would be a useful genetic tool for analysing the function of genes or for the production of biofuel or biomaterials.

In this study, we introduced a strong SD-like sequence to enhance gene expression. However, in biofuel production applications, different RBSs of various strengths would be helpful for optimizing the expression levels of the enzymes in the biosynthetic pathway to avoid undesired byproducts. A series of SD-like sequences ranging in strength may be conveniently designed with the help of a software developed by Salis and colleagues (2009) that predicts RBS strength in cyanobacteria.

The ultimate goal is to develop a light-dependent feasible gene expression system for biofuel and/or biomaterials, which require multiple genes for their synthesis. The current status of our achievement is to regulate a single gene product expression by green light. However, even single gene expression, the regulatory gene, transcriptional factor and/or gene product affecting the cell viability will be regulated, which in consequent, may affect the biofuel and/or biomaterial production level.

In conclusion, we characterized a green-light sensing system consisting of *ccaS*, *ccaR*, and the target promoter P_cpcG2_ in *Synechocystis* sp. PCC6803. We found this system activated target gene expression from P_cpcG2_ by illumination with green light even when simultaneously illuminated with red light. Gene expression in cyanobacteria can therefore be effectively regulated by green light while maintaining adequate growth conditions with red light. The green-light sensing system was further engineered by enhancing the CcaR expression level and by inserting an SD-like sequence, achieving a 40-fold increase in target gene expression level. This expression level is comparable with the levels obtained with P_trc_, which is one of the strongest promoters used in recombinant DNA experiments with cyanobacteria. The engineered green-light gene expression system would be a useful genetic tool for controlling gene expression in the emergent cyanobacterial bioprocesses.

## Supporting information

Table S1 Sequence of the DNAinserted into the PstI sites of pKT230 (replacing the smaller fragment) for each genetic construct.
