# Supplementary material for: Engineering of a green-light inducible gene expression system in Synechocystis sp. PCC6803
Source: Microb Biotechnol. 2013 Dec 12;7(2):177–83. doi: 10.1111/1751-7915.12098 (PMC3937721; doi:10.1111/1751-7915.12098)
Supplement: Table S1 — Sequence of the DNAinserted into the PstI sites of pKT230 (replacing the smaller fragment) for each genetic construct. [file mbt20007-0177-sd1.docx]

**Supplementary information**

Table S1. Sequence of the DNA inserted into the PstI sites of pKT230 (replacing the smaller fragment) for each genetic construct.

| Name | Sequence^*^ |
| --- | --- |
| P_cpcG2_-*GFPuv* | CTGCAGtcctccactaaaagaattctcatagcccattgtgcttttctctatcaacctcagcttacctgaaggggtgaacaggtctgggttaattcatgttgcgaaatgtaacagttttagtcgcatcagctaactttccgatttctttacgattttctcccccttttcttcaattttactttgttaggatcgcatttttaatgccaacacataccagttattggctggacattaaacaacttttaagtttaattactaactttacatatgagtaaaggagaagaacttttcactggagttgtcccaattcttgttgaattagatggtgatgttaatgggcacaaattttctgtcagtggagagggtgaaggtgatgcaacatacggaaaacttacccttaaatttatttgcactactggaaaactacctgttccatggccaacacttgtcactactttctcttatggtgttcaatgcttttcccgttatccggatcacatgaaacggcatgactttttcaagagtgccatgcccgaaggttatgtacaggaacgcactatatctttcaaagatgacgggaactacaagacgcgtgctgaagtcaagtttgaaggtgatacccttgttaatcgtatcgagttaaaaggtattgattttaaagaagatggaaacattctcggacacaaactcgagtacaactataactcacacaatgtatacatcacggcagacaaacaaaagaatggaatcaaagctaacttcaaaattcgccacaacattgaagatggatccgttcaactagcagaccattatcaacaaaatactccaattggcgatggccctgtccttttaccagacaaccattacctgtcgacacaatctgccctttcgaaagatcccaacgaaaagcgtgaccacatggtccttcttgagtttgtaactgctgctgggattacacatggcatggatgagctctacaaataatctagatttttttgacggtaaagccaagctgaaggctgacaaaatctatactgttatatccccctttcctgggggacttaggcggtttaataactggggtttttgctagaaatattttggcgtttaattatttattctctggtttaatatttcgagactttgcagactttgtatttgaggccagactccaatgttgaaactagccggtaaaaaagcctctgccctgacctgcaagaaaacgaaggcaaaaattggtatcctagaaatggatcggcagtggtatcaatcagtcaagcgatttttatgtccaagttttgaaatttctgctatcaacttccaagatattttgtttaacaatgttgacgttggagaatatcattcgatcctagttggttgcgggattaaatatgaatcaattgatatttcagctactttagatttagtgaccattattaaaaaattgtctgctaagccgccggccctactactaattacggattgtgccgacagtcagatcacggtacaagctcgcagttacctgccccaaattgacggcgtttttgccaaggatcatgatttagctttattgttaaaagtcatgaaaattattgccaaacaaaaatattttaagtaaggtttttctactgggattcgctaatatCTGCAG |
| P_cpcG2_-*GFPuv*-P_ccaS_-*ccaS* | CTGCAGtcctccactaaaagaattctcatagcccattgtgcttttctctatcaacctcagcttacctgaaggggtgaacaggtctgggttaattcatgttgcgaaatgtaacagttttagtcgcatcagctaactttccgatttctttacgattttctcccccttttcttcaattttactttgttaggatcgcatttttaatgccaacacataccagttattggctggacattaaacaacttttaagtttaattactaactttacatatgagtaaaggagaagaacttttcactggagttgtcccaattcttgttgaattagatggtgatgttaatgggcacaaattttctgtcagtggagagggtgaaggtgatgcaacatacggaaaacttacccttaaatttatttgcactactggaaaactacctgttccatggccaacacttgtcactactttctcttatggtgttcaatgcttttcccgttatccggatcacatgaaacggcatgactttttcaagagtgccatgcccgaaggttatgtacaggaacgcactatatctttcaaagatgacgggaactacaagacgcgtgctgaagtcaagtttgaaggtgatacccttgttaatcgtatcgagttaaaaggtattgattttaaagaagatggaaacattctcggacacaaactcgagtacaactataactcacacaatgtatacatcacggcagacaaacaaaagaatggaatcaaagctaacttcaaaattcgccacaacattgaagatggatccgttcaactagcagaccattatcaacaaaatactccaattggcgatggccctgtccttttaccagacaaccattacctgtcgacacaatctgccctttcgaaagatcccaacgaaaagcgtgaccacatggtccttcttgagtttgtaactgctgctgggattacacatggcatggatgagctctacaaataatctagatttttttgacggtaaagccaagctgaaggctgacaaaatctatactgttatatccccctttcctgggggacttaggcggtttaataactggggtttttgctagaaatattttggcgtttaattatttattctctggtttaatatttcgagactttgcagactttgtatttgaggccagactccaatgttgaaactagccggtaaaaaagcctctgccctgacctgcaagaaaacgaaggcaaaaattggtatcctagaaatggatcggcagtggtatcaatcagtcaagcgatttttatgtccaagttttgaaatttctgctatcaacttccaagatattttgtttaacaatgttgacgttggagaatatcattcgatcctagttggttgcgggattaaatatgaatcaattgatatttcagctactttagatttagtgaccattattaaaaaattgtctgctaagccgccggccctactactaattacggattgtgccgacagtcagatcacggtacaagctcgcagttacctgccccaaattgacggcgtttttgccaaggatcatgatttagctttattgttaaaagtcatgaaaattattgccaaacaaaaatattttaagtaaggtttttctactgggattcgctaatatatgggcaaatttctaattccaatcgaatttgtttttctggcgatcgccatgacctgttatttatggcacagacaaaaccaagaacgccgcaggattgaaattagcatcaagcaacaaacccaacgggaacgatttattaaccaaattacccaacatatccgccaatctttaaacttggaaacggttttaaataccaccgtcgctgaagttaaaaccctgttgcaagttgatcgagttctaatttatcgcatttggcaagatggcacgggcagcgccattacggaatcggtgaatgccaattatcctagtattttagggcggaccttttccgatgaagtttttcccgttgaataccatcaagcctacaccaaaggtaaagtacgggccattaatgacattgaccaggatgacatagagatttgcctagctgatttcgtcaaacaatttggcgtgaaatcaaaattagtagtgcccattcttcaacataatcgtgcttcttccctagataatgaatcagaatttccctatctttgggggctgttaattacccatcaatgtgcttttacccggccatggcaaccgtgggaagtggagttaatgaaacagctagccaatcaggtcgcgatcgccatccaacaatcggaattatatgagcaattacagcaactcaataaagatttggaaaaccgagtcgaaaaacgcacccagcaacttgccgccaccaatcaatccctaagaatggaaatcagtgagcgacaaaaaacggaagccgctctccgccacactaaccatactctgcaatccctgattgcggcctcccccaggggtatttttacccttaatttagcagaccaaattcagatttggaatcctacagcagaacgtatttttggttggacagaaacagaaattattgcccatccagaattattaacatccaacattttgctggaagattatcagcaatttaaacagaaagttttatcaggcatggtttcccctagcctagaattaaaatgtcaaaaaaaagatggtagttggattgaaattgtcctttccgctgctcccctattggatagtgaagaaaatattgccggattggtggcggttgtcgccgatattaccgagcaaaagcggcaggcagaacaaattcgtttgctacaatccgttgtggttaatactaatgatgcggtggtgattacggaagcggagcccattgatgatcccgggccgagaattctctatgtcaatgaagcatttactaaaatcaccggttatactgctgaagaaatgctaggcaaaaccccccgagttttacagggaccaaaaactagtcgcactgaattagatagggtgcggcaagccattagtcaatggcaatcagttaccgttgaagtgattaattatcgtaaggatggcagtgagttttgggtggaatttagtctggtgcccgttgccaataaaacaggtttttacacccattggattgctgtgcaaagggatgtcactgagcgccgacgcacggaggaagtccgcctagctttagaacgggaaaaagaattaagccgcctaaaaactcgttttttctccatggcttcccatgaatttcgtactcccctcagtacggccttagctgctgcccaattactggaaaattctgaagtggcctggcttgatcccgataagcgtagccggaacttacaccgtattcaaaattccgtgaaaaatatggtacagctcctggatgatattttaatcattaaccgtgccgaagcgggcaaattggaatttaatcctaattggttagatttgaaattattgttccagcaatttatcgaagaaattcaattaagtgtcagtgaccaatattattttgactttatttgtagcgctcaagatacgaaggcattggtggatgaaaggttagtgcggtctattttatctaatctgttatctaatgcgattaaatactctcccgggggagggcagattaaaattgccctaagcctagattcggaacagattatttttgaagtcaccgaccagggcattggcatttcgccagaggaccaaaagcaaatttttgaaccctttcatcggggcaaaaatgtcagaaatattacgggaacaggactcggtttaatggttgccaagaaatgtgttgacttacacagtggcagtatcttgctaaaaagtgcagttgaccagggaacaacagttactatctgtttaaaacgctataaccatttgcctcgagcttagcgcaaaaaaccccgcttcggcggggttttttcgcCTGCAG |
| P_cpcG2_-*GFPuv*-P_ccaR_-*ccaR* | CTGCAGtcctccactaaaagaattctcataggagagcgttcaccgacaaacaacagataaaacgaaaggcccagtctttcgactgagcctttcgttttatttgatgcctggcagttccctactctcgcatggggagaccccacactaccatcggcgctacggcgtttcacttctgagttcggcatggggtcaggtgggaccaccgcgctactgccgccaggcaaattctgttttatcagaccgcttctgcgttctgatttaatctgtatcaggctgaaaatcttctctcatccgccaaaacagccaagcttctagtttttcccttggcacaaagatttttccgttaaattggccagacgatacccaatgccatggaccgtttcaatggcatctgctgataaaccggcactttttaatttttgtcgcaaactccgcacatgcaccttaaccgtatcttcctctgggggactctccaacttccagatactatcgataatcatgctccgacttagcacccgacggccattgcggagtagtaattccagaatgctgtattccttgcgggtcaaagacaaaacctcattgtcataactaacttcataggtgcttggatccaacctgattggcccccactctaaaactggttggcacgttgcacaaccccgacgcaacaaagctcgcaccctggcaaataactcccccaaatccactggcttgaccacataatcatccgcccccgcatccaagcccgtgattttatcattgatcgtatccctggctgtcatcatcaaaattggcattaaataactgtgcgatcgccatttttgacagagggtaatcccgtccaactccggcagcatcacatccaaaataacgaggtcatattccagtcgggaggcatagtcccaggcgagggaagcgtcggtggcaatatcaacggtgtaaagctggtcactcaatgcttcagcaagggtttccgccagcggcaaatcatcctccactaaaagaattctcatagcccattgtgcttttctctatcaacctcagcttacctgaaggggtgaacaggtctgggttaattcatgttgcgaaatgtaacagttttagtcgcatcagctaactttccgatttctttacgattttctcccccttttcttcaattttactttgttaggatcgcatttttaatgccaacacataccagttattggctggacattaaacaacttttaagtttaattactaactttacatatgagtaaaggagaagaacttttcactggagttgtcccaattcttgttgaattagatggtgatgttaatgggcacaaattttctgtcagtggagagggtgaaggtgatgcaacatacggaaaacttacccttaaatttatttgcactactggaaaactacctgttccatggccaacacttgtcactactttctcttatggtgttcaatgcttttcccgttatccggatcacatgaaacggcatgactttttcaagagtgccatgcccgaaggttatgtacaggaacgcactatatctttcaaagatgacgggaactacaagacgcgtgctgaagtcaagtttgaaggtgatacccttgttaatcgtatcgagttaaaaggtattgattttaaagaagatggaaacattctcggacacaaactcgagtacaactataactcacacaatgtatacatcacggcagacaaacaaaagaatggaatcaaagctaacttcaaaattcgccacaacattgaagatggatccgttcaactagcagaccattatcaacaaaatactccaattggcgatggccctgtccttttaccagacaaccattacctgtcgacacaatctgccctttcgaaagatcccaacgaaaagcgtgaccacatggtccttcttgagtttgtaactgctgctgggattacacatggcatggatgagctctacaaataatctagatttttttgacggtaaagccaagctgaaggctgacaaaatctatactgttatatccccctttcctgggggacttaggcggtttaataactggggtttttgctagaaatattttggcgtttaattatttattctctggtttaatatttcgagactttgcagactttgtatttgaggccagactccaatgttgaaactagccggtaaaaaagcctctgccctgacctgcaagaaaacgaaggcaaaaattggtatcctagaaatggatcggcagtggtatcaatcagtcaagcgatttttatgtccaagttttgaaatttctgctatcaacttccaagatattttgtttaacaatgttgacgttggagaatatcattcgatcctagttggttgcgggattaaatatgaatcaattgatatttcagctactttagatttagtgaccattattaaaaaattgtctgctaagccgccggccctactactaattacggattgtgccgacagtcagatcacggtacaagctcgcagttacctgccccaaattgacggcgtttttgccaaggatcatgatttagctttattgttaaaagtcatgaaaattattgccaaacaaaaatattttaagtaaggtttttctactgggattcgctaatatCTGCAG |
| PcpcG2-SD-GFPuv^**^ | CTGCAGtcctccactaaaagaattctcatagcccattgtgcttttctctatcaacctcagcttacctgaaggggtgaacaggtctgggttaattcatgttgcgaaatgtaacagttttagtcgcatcagctaactttccgatttctttacgattttctcccccttttcttcaattttactttgttaggatcgcatttttaatgccaacacataccagttattggctggacattaaacaacttttaagtttaattactaactttacatataagtaggagataaaaatatgagtaaaggagaagaacttttcactggagttgtcccaattcttgttgaattagatggtgatgttaatgggcacaaattttctgtcagtggagagggtgaaggtgatgcaacatacggaaaacttacccttaaatttatttgcactactggaaaactacctgttccatggccaacacttgtcactactttctcttatggtgttcaatgcttttcccgttatccggatcacatgaaacggcatgactttttcaagagtgccatgcccgaaggttatgtacaggaacgcactatatctttcaaagatgacgggaactacaagacgcgtgctgaagtcaagtttgaaggtgatacccttgttaatcgtatcgagttaaaaggtattgattttaaagaagatggaaacattctcggacacaaactcgagtacaactataactcacacaatgtatacatcacggcagacaaacaaaagaatggaatcaaagctaacttcaaaattcgccacaacattgaagatggatccgttcaactagcagaccattatcaacaaaatactccaattggcgatggccctgtccttttaccagacaaccattacctgtcgacacaatctgccctttcgaaagatcccaacgaaaagcgtgaccacatggtccttcttgagtttgtaactgctgctgggattacacatggcatggatgagctctacaaataaTCTAGAtttttttgacggtaaagccaagctgaaggctgacaaaatctatactgttatatccccctttcctgggggacttaggcggtttaataactggggtttttgctagaaatattttggcgtttaattatttattctctggtttaatatttcgagactttgcagactttgtatttgaggccagactccaatgttgaaactagccggtaaaaaagcctctgccctgacctgcaagaaaacgaaggcaaaaattggtatcctagaaatggatcggcagtggtatcaatcagtcaagcgatttttatgtccaagttttgaaatttctgctatcaacttccaagatattttgtttaacaatgttgacgttggagaatatcattcgatcctagttggttgcgggattaaatatgaatcaattgatatttcagctactttagatttagtgaccattattaaaaaattgtctgctaagccgccggccctactactaattacggattgtgccgacagtcagatcacggtacaagctcgcagttacctgccccaaattgacggcgtttttgccaaggatcatgatttagctttattgttaaaagtcatgaaaattattgccaaacaaaaatattttaagtaaggtttttctactgggattcgctaatatCTGCAG |
| PcpcG2-SD-GFPuv+P_ccaR-_*ccaR* | CTGCAGtcctccactaaaagaattctcataggagagcgttcaccgacaaacaacagataaaacgaaaggcccagtctttcgactgagcctttcgttttatttgatgcctggcagttccctactctcgcatggggagaccccacactaccatcggcgctacggcgtttcacttctgagttcggcatggggtcaggtgggaccaccgcgctactgccgccaggcaaattctgttttatcagaccgcttctgcgttctgatttaatctgtatcaggctgaaaatcttctctcatccgccaaaacagccaagcttctagtttttcccttggcacaaagatttttccgttaaattggccagacgatacccaatgccatggaccgtttcaatggcatctgctgataaaccggcactttttaatttttgtcgcaaactccgcacatgcaccttaaccgtatcttcctctgggggactctccaacttccagatactatcgataatcatgctccgacttagcacccgacggccattgcggagtagtaattccagaatgctgtattccttgcgggtcaaagacaaaacctcattgtcataactaacttcataggtgcttggatccaacctgattggcccccactctaaaactggttggcacgttgcacaaccccgacgcaacaaagctcgcaccctggcaaataactcccccaaatccactggcttgaccacataatcatccgcccccgcatccaagcccgtgattttatcattgatcgtatccctggctgtcatcatcaaaattggcattaaataactgtgcgatcgccatttttgacagagggtaatcccgtccaactccggcagcatcacatccaaaataacgaggtcatattccagtcgggaggcatagtcccaggcgagggaagcgtcggtggcaatatcaacggtgtaaagctggtcactcaatgcttcagcaagggtttccgccagcggcaaatcatcctccactaaaagaattctcatagcccattgtgcttttctctatcaacctcagcttacctgaaggggtgaacaggtctgggttaattcatgttgcgaaatgtaacagttttagtcgcatcagctaactttccgatttctttacgattttctcccccttttcttcaattttactttgttaggatcgcatttttaatgccaacacataccagttattggctggacattaaacaacttttaagtttaattactaactttacatataagtaggagataaaaatatgagtaaaggagaagaacttttcactggagttgtcccaattcttgttgaattagatggtgatgttaatgggcacaaattttctgtcagtggagagggtgaaggtgatgcaacatacggaaaacttacccttaaatttatttgcactactggaaaactacctgttccatggccaacacttgtcactactttctcttatggtgttcaatgcttttcccgttatccggatcacatgaaacggcatgactttttcaagagtgccatgcccgaaggttatgtacaggaacgcactatatctttcaaagatgacgggaactacaagacgcgtgctgaagtcaagtttgaaggtgatacccttgttaatcgtatcgagttaaaaggtattgattttaaagaagatggaaacattctcggacacaaactcgagtacaactataactcacacaatgtatacatcacggcagacaaacaaaagaatggaatcaaagctaacttcaaaattcgccacaacattgaagatggatccgttcaactagcagaccattatcaacaaaatactccaattggcgatggccctgtccttttaccagacaaccattacctgtcgacacaatctgccctttcgaaagatcccaacgaaaagcgtgaccacatggtccttcttgagtttgtaactgctgctgggattacacatggcatggatgagctctacaaataatctagatttttttgacggtaaagccaagctgaaggctgacaaaatctatactgttatatccccctttcctgggggacttaggcggtttaataactggggtttttgctagaaatattttggcgtttaattatttattctctggtttaatatttcgagactttgcagactttgtatttgaggccagactccaatgttgaaactagccggtaaaaaagcctctgccctgacctgcaagaaaacgaaggcaaaaattggtatcctagaaatggatcggcagtggtatcaatcagtcaagcgatttttatgtccaagttttgaaatttctgctatcaacttccaagatattttgtttaacaatgttgacgttggagaatatcattcgatcctagttggttgcgggattaaatatgaatcaattgatatttcagctactttagatttagtgaccattattaaaaaattgtctgctaagccgccggccctactactaattacggattgtgccgacagtcagatcacggtacaagctcgcagttacctgccccaaattgacggcgtttttgccaaggatcatgatttagctttattgttaaaagtcatgaaaattattgccaaacaaaaatattttaagtaaggtttttctactgggattcgctaatatCTGCAG |

*The regions encoding GFPuv (green), CcaS (light blue), and CcaR (yellow) are highlighted.

** The sequence derived from the *cpcB* gene of *Synechococcus* sp. PCC7002 containing the SD-like sequence is in blue letters. This sequence was added by ligating synthetic double-stranded DNA having 5′ TA overhangs into the *Nde*I site at the start codon of GFPuv. This results in a cytosine to thymine substitution, shown in white letter over a black background.
